# Steroid therapy is linked to lower incidence of acute kidney injury in patients with severe alcohol-associated hepatitis

**DOI:** 10.1038/s41598-025-29912-4

**Published:** 2025-12-08

**Authors:** Laura Buttler, Jan Stange, Nikolaos Pyrsopoulos, Tarek Hassanein, Heiner Wedemeyer, Benjamin Maasoumy, Markus Busch

**Affiliations:** 1 Department of Gastroenterology, Hepatology, Infectious Diseases and Endocrinology, Hannover Medical School, Carl-Neuberg-Str.1, 30625 Hannover, Germany; 2 Center for Extracorporeal Organ Support (CEOS), Department of Nephrology, Biomedical Research Center, University of Rostock, Rostock, Germany; 3 Liver Disease in New Jersey, NYU Grossman School of Medicine, NYU Langone Transplant Institute, New York, USA; 4 Southern California Research Center, Coronado, CA USA; 5 German Center for Infection Research (DZIF), Hannover-Braunschweig, Germany; 6 Vital Therapies, Inc., San Diego, USA; 7 Drexel University, Philadelphia, USA; 8 Piedmont Atlanta Hospital, Atlanta, USA; 9 Rutgers University Hospital, Newark, USA; 10 Hospital Universitario Puerta de Hierro Majadahonda, Majadahonda, Spain; 11 Beth Israel Deaconess Medical Center, Boston, USA; 12 Rush University Medical Center, Chicago, USA; 13 Albert Einstein Medical Center, Philadelphia, USA; 14 Hospital Clinic de Barcelona, Barcelona, Spain; 15 Emory University Hospital, Atlanta, USA; 16 University of Arkansas for Medical Sciences, Little Rock, USA; 17 University of Minnesota, Minneapolis, USA; 18 Universitätsmedizin Mainz, Mainz, Germany; 19 Universitätsklinikum Münster, Münster, Germany; 20 Medizinische Hochschule Hannover, Hannover, Germany; 21 Montefiore Medical Center, The Bronx, USA; 22 Cleveland Clinic Foundation, Cleveland, USA; 23 Houston Methodist Hospital, Houston, USA; 24 Aurora St. Luke’s Medical Center, Milwaukee, USA; 25 University of Virginia Health System, Charlottesville, USA; 26 University of Pittsburgh Medical Center, Pittsburgh, USA; 27 Schiff Center for Liver Diseases/University of Miami, Coral Gables, USA; 28 Hospital Universitario Virgen del Rocío, Seville, Spain; 29 Sharp Coronado Hospital, Coronado, USA; 30 Stanford University Medical Center, Stanford, USA; 31 Hospital Universitario y Politécnico La Fe, Valencia, Spain; 32 Hospital Universitario Gregorio Marañón, Madrid, Spain; 33 University of Missouri Hospital, Columbia, USA; 34 Klinikum Landshut Gemeinnuetzige GmbH, Landshut, Germany; 35 The Pennsylvania State University and The Milton S. Hershey Medical Center, Hershey, USA; 36 Medizinische Universität Graz, Graz, Austria; 37 Medizinische Universität Klinik Für Innere Medizin III, Salzburg, Austria; 38 Universitätsmedizin Rostock, Rostock, Germany; 39 Mercy Medical Center, Baltimore, USA; 40 Hospital Clínico Universitario Lozano Blesa, Zaragoza, Spain

**Keywords:** Severe alcohol-associated hepatitis (sAH), Acute kidney disease (AKI), Renal complications in liver disease, Inflammation-driven AKI, Corticosteroids in alcohol-associated hepatitis, Diseases, Medical research, Nephrology, Risk factors

## Abstract

Acute kidney injury (AKI) is a common complication in patients with severe alcohol-associated hepatitis (sAH), yet its risk factors and outcomes remain incompletely defined. Corticosteroids are the only established therapy for sAH, but their impact on renal outcomes is unknown. A post-hoc analysis of the prospective multicenter VTL-308 trial including 151 patients with sAH was conducted to evaluate the incidence and predictors of AKI, the association of corticosteroid therapy with AKI risk, and factors associated with AKI reversal during 90 days of follow-up. AKI was independently associated with significantly higher 90-day mortality (subdistribution hazard ratio (sHR) = 8.74; p < 0.001). In multivariate competing risk analysis, higher bilirubin levels (sHR = 1.06; p = 0.003) were linked to increased AKI risk, while corticosteroids were associated with an ameliorated AKI risk (sHR = 0.47; p = 0.01). Time-censored analyses confirmed the protective association of corticosteroids (HR = 0.25; p = 0.001). Once AKI occurred, corticosteroids did not promote renal recovery (HR = 1.15; p = 0.74). Elevated bilirubin levels were linked to a lower probability of AKI reversal. In conclusion, AKI is a devastating complication in sAH. Corticosteroid therapy is independently associated with a significantly lower risk of AKI development but does not influence recovery once AKI has occurred. Future prospective trials are required to provide further validation of these findings.

**Supplementary Information:**

The online version contains supplementary material available at 10.1038/s41598-025-29912-4.

## Introduction

Severe alcohol-associated hepatitis (sAH) is a rapidly progressive inflammatory liver disease triggered by recent heavy alcohol use in patients with relatively mild underlying chronic liver disease^1^. Its incidence is rising globally, particularly among younger individuals and women^2^. Despite intensive care, the 90-day mortality rate for AH remains high ranging from 23 to 29%^3,4^, and complications such as acute kidney injury (AKI) emerge as key determinants of outcome^5–7^.

AKI occurs frequently in patients with liver disease and represents a major clinical challenge in the management of decompensated cirrhosis (DC) and acute-on-chronic liver failure (ACLF)^8–10^. In these syndromes, renal dysfunction is a well-established predictor of mortality. However, compared to DC and ACLF, the incidence, risk factors, and clinical implications of AKI in sAH remain less well characterized.

Given that sAH may occur independently of cirrhosis and is marked by profound systemic inflammation, the pathophysiology of AKI in this context may differ substantially from other forms of liver-related kidney injury. Understanding specific risk and protective factors for AKI in sAH is therefore crucial to identify modifiable targets, guide clinical management and improve patient outcomes.

In this study, we aimed to (i) determine the incidence and mortality of AKI, (ii) investigate predictors of AKI development, (iii) evaluate the impact of corticosteroid therapy on AKI risk, and (iv) identify factors associated with AKI reversal in a well-defined cohort of patients with sAH.

## Methods

### Study population

A post hoc analysis of the multicenter, randomized, open-label VTL-308 trial, which was conducted between 2016 and 2018, was performed^11,12^. In this study, 151 patients with sAH were included and randomized to receive either standard of care (SOC) treatment plus Extracorporeal Liver Assist Device (ELAD) therapy (ELAD group) or SOC alone (control group), as described previously^12^. The ELAD system is a hepatic cell-based liver support device, consisting of four cartridges, containing cloned, immortalized human hepatoblastoma cells (VTL C3A cells). Through a dual-lumen central venous catheter blood was withdrawn, which was subsequently separated into cellular components and ultrafiltrate. The latter circulated through the cartridges before being recombined and reinfused into the patient^12^. However, ELAD therapy, was not linked to a significant improvement on patients` outcomes. According to the VTL-308 protocol, the decision for corticosteroid use was left to the respective treating investigators and administration followed the Lille model, with continuation determined after seven days based on bilirubin response^12^. Since patients were recruited as part of a clinical trial evaluating the efficacy of a liver support system designed to bridge individuals with sAH through the acute phase of liver failure, the key inclusion criteria required the absence of extrahepatic organ failure, including chronic end-stage kidney injury requiring hemodialysis for a time period longer than eight weeks and subjects on hemodialysis, and specifically excluded patients with advanced cirrhosis. The rationale was to enroll individuals with high regenerative potential and relatively mild underlying chronic liver disease and to exclude those with end-stage disease unlikely to recover. Detailed inclusion and exclusion criteria are provided in Supplementary Table 1. Importantly, administration of corticosteroids was not part of the VTL-308 study protocol and was left to the respective investigators´ discretion^12^. Prospective data collection took place during hospitalization and through weekly home visits after hospital discharge^12^. Baseline was defined as the time point of VTL-308 study inclusion, not as time point of hospital admission. Patients were observed for a period of 90 days and were censored in case of loss to follow-up.

### Definition of sAH

Patients were diagnosed with sAH in a two-step process: first, based on recent alcohol use (≤ 6 weeks before admission) consistent with alcohol-induced liver decompensation (AILD); second, by fulfilling either histological confirmation or ≥ 2 typical clinical features (hepatomegaly, AST > ALT, ascites, leukocytosis), alongside a bilirubin ≥ 16 mg/dl and a Maddrey discriminant function (DF) score ≥ 32^12^.

### Definition of AKI

AKI and its staging were defined according to the criteria established by the International Club of Ascites (ICA)^13^. The diagnosis of AKI was based on an acute increase of serum creatinine (sCr) ≥ 0.3 mg/dl (≥ 26.5 μmol/l) from baseline within 48 h, or a ≥ 50% increase in sCr from a known value within the prior seven days.

AKI was staged as follows:

- Stage 1: Increase in sCr of ≥ 0.3 mg/dl (≥ 26.5 μmol/l) or 1.5–twofold from baseline
- Stage 2: Increase in sCr of > 2–threefold from baseline
- Stage 3: Increase in sCr of > threefold from baseline or sCr ≥ 4.0 mg/dl (≥ 353.6 μmol/l) with an acute increase of at least 0.3 mg/dl (26.5 μmol/l) or initiation of renal replacement therapy.

For subsequent analyses, AKI was evaluated as a binary variable (presence vs. absence), irrespective of the AKI grade.

For AKI reversal, a full response according to the International Club of Ascites criteria with a return of creatinine concentrations within 0.3 mg/dl of the baseline value was required^13^. In cases where creatinine values were not available, information about recovery from AKI was provided by the respective treating investigators.

### Statistical approach

We investigated four main aspects:

(i) Impact of AKI on mortality,
(ii) Predictors of AKI development,
(iii) Impact of corticosteroid therapy on AKI incidence, and
(iv) Factors associated with AKI reversal.

(i) Impact of AKI on mortality

The association between AKI and 90-day mortality was analyzed using two statistical approaches: A competing risk regression model was used, treating liver transplantation (LTx) as competing event. A Cox proportional hazard regression with AKI treated as a time-dependent covariate. Patients were assigned to the AKI-group only after AKI onset. In multivariate models, AKI was the primary variable of interest. The other covariate GAHS was included based on clinical relevance and prior evidence. Results were reported as subdistribution hazard ratios (sHR) for competing risk models and hazard ratios (HR) for Cox models, each with 95% confidence intervals (CIs).

(ii) Predictors of AKI development

To identify factors independently associated with the development of AKI, we analyzed 140 patients without AKI at baseline (eleven patients with AKI at baseline were excluded). A competing risk regression model was used with AKI as endpoint, treating LTx and death as competing events. The competing risk analyses were performed univariate and multivariate. Variables with significant associations in univariate analysis (p < 0.05) were entered into multivariate models. To avoid collinearity, composite scores (e.g., MELD, Maddrey’s) and their component laboratory values were not included simultaneously. Final models included bilirubin and corticosteroid therapy as key variables based on clinical relevance and statistical significance.

(iii) Impact of corticosteroid therapy on AKI incidence

The specific impact of corticosteroid therapy on AKI risk was assessed using two complementary competing risk models: 1. Patients were stratified based on baseline corticosteroid use into ‘steroid’ and ‘no steroid’ groups. 2. A time-censored competing risk model was applied to account for changes in steroid exposure during follow up: patients in the steroid group were censored at the time of steroid discontinuation, and patients in the no-steroid group were censored at the time of steroid initiation. Both models treated LTx and death as competing events and were initially analyzed univariately, followed by multivariate adjustment for baseline bilirubin levels, which had been independently associated with AKI risk in prior analyses. To reduce potential confounding, the statistical approach was augmented by a matched analysis, in which patients with corticosteroid treatment were matched to those without in a 1:1 ratio, incorporating the matching variables sex and GAHS. Matching was performed using nearest neighbor matching based on propensity score with a caliper of 0.25 and was considered to be successful when reaching standardized mean differences between -0.1 and 0.1. Respecting these criteria, 53 patients receiving steroid treatment were matched with 53 patients without steroid intake. Details are displayed in the Supplementary tables 2–3 and in Supplementary Fig. 1.

(iv) Factors associated with AKI reversal

Among patients who developed AKI, factors associated with renal recovery were investigated using Cox regression models. The association between corticosteroid therapy, bilirubin levels, albumin therapy, and other clinical and laboratory parameters (WBC, INR, presence of cirrhosis) and AKI reversal was assessed.

**Fig. 1:**
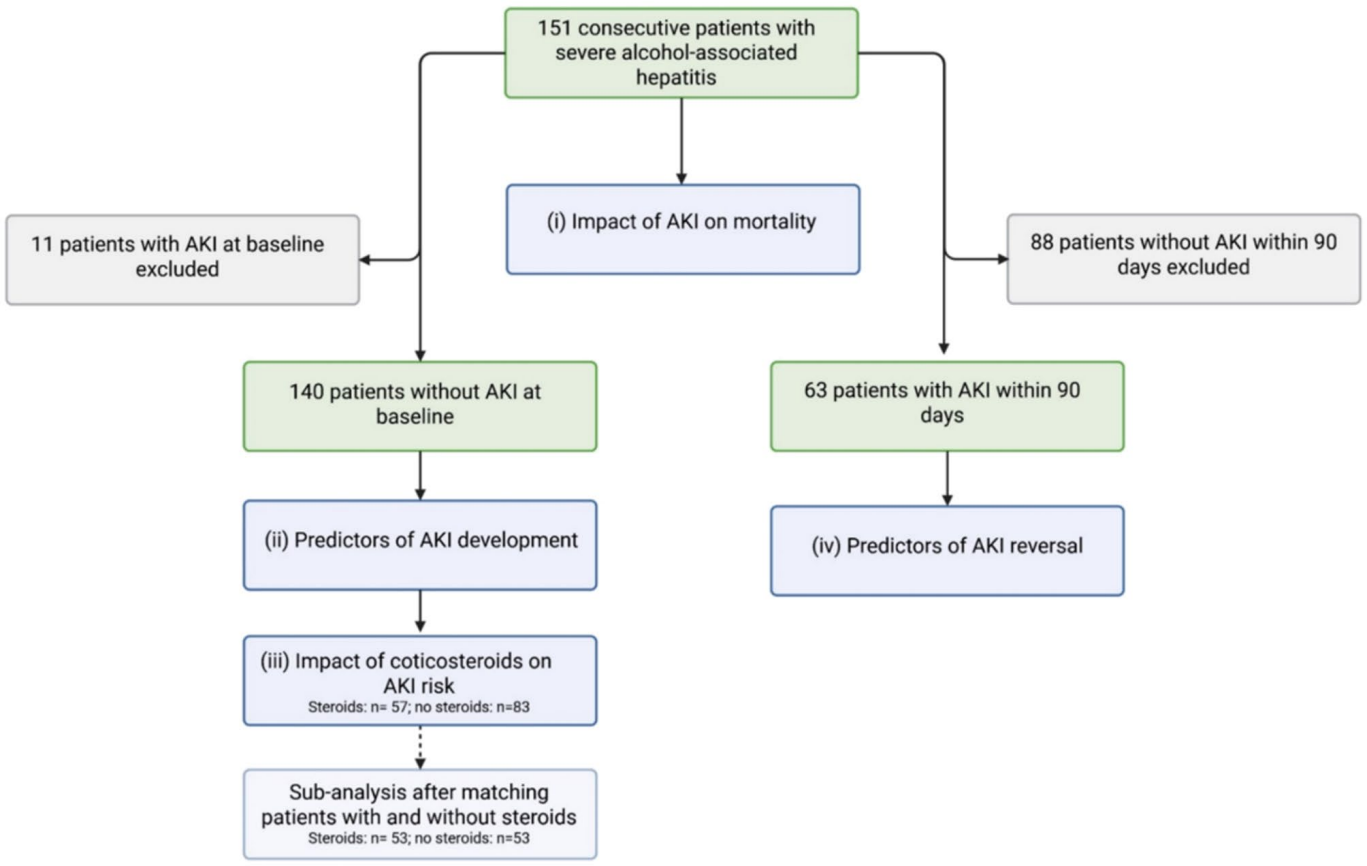
Study flow chart. Figure 1 provides a summary of the included patients and the conducted analyses. AKI: Acute kidney injury.

A summary of all analyses is provided in Fig. 1.

### Ethics approval

The study (NCT02612428) was approved by the ethics committees of the VTL-308 study centers and adhered to the Declarations of Helsinki. Written informed consent was required for inclusion.

### Statistics

Patient characteristics were summarized using descriptive statistics, analyzed with R Statistical Software (version 4.2.0, R Foundation for Statistical Computing, Vienna, Austria) with the “tableone” package and IBM SPSS Statistics (Version 28, IBM®, New York). Categorial parameters were displayed as number and percentages and compared with the chi-square or Fisher’s exact test, as appropriate. Medians and interquartile ranges (IQRs) were used to describe continuous values, which were compared with the Mann–Whitney U test due to non-normal distribution. McNemar and Wilcoxon test were used for the comparison of categorial and continuous variables after matching, respectively. Cox regression and competing risk analyses were conducted in Rcmdr with the EZR plugin. A p-value < 0.05 was considered statistically significant. Propensity score matching was performed using R studio with the “MatchIt” package.

## Results

### Cohort characterization

A total of 151 patients with sAH were included in this study. The median age was 40 years, and 60% of the cohort were male. The median MELD score at baseline was 25, and the median Maddrey Discriminant Function (MDF) was 63. Eleven patients (15.2%) presented with AKI at baseline and were excluded from further analyses. Regarding comorbidities, around 70% of patients had pre-existing compensated liver cirrhosis, and approximately 11% had diabetes mellitus. The follow-up period lasted 90 days, during which 63 patients (41.7%) developed AKI, 4 patients (2.6%) underwent LTx, and 31 patients (20.5%) died. In patients with corticosteroid treatment, median baseline dose was 40.0 mg prednisolone or methylprednisolone daily, which was comparable between patients of the AKI and no AKI group (p = 0.64). Regarding the 57 patients who were treated with prednisolone, the majority (n = 52; 91.2%) received 40 mg prednisolone daily. A minor proportion (n = 2; 3.5%) was treated with 30 mg per day. 10 mg, 45 mg and 50 mg were administered to only one patient (1.8%), respectively. A number of six patients received methylprednisolone, with 40 mg in four patients (66.7%) and 32 mg and 10 mg in one patient (16.7%), respectively. Baseline clinical and laboratory characteristics, including organ dysfunction and relevant scoring systems, are summarized in Table 1.

**Table 1:** Baseline characteristics. This table displays the baseline characteristics of the studied patients at the time point of study inclusion. Categorial parameters are shown as number and percentages and were compared using the chi-square test, continuous parameters are described as medians and interquartile ranges (IQRs) and were compared with the Mann–Whitney U test. ALT: Alanine aminotransferase, AP: Alkaline phosphatase, AST: Aspartate aminotransferase, BL: Baseline, ELAD: Extracorporal Liver Assist Device, GAHS: Glasgow alcoholic hepatitis score, INR: International normalized ratio, MELD: Model for End-Stage Liver Disease, Y: Years.

	No steroids	Steroids	p value*
Number	88 (58.3)	63 (41.7)	
Male sex	52 (59.1)	39 (61.9)	0.86
Age (y)	42.0 (35.0, 46.0)	39.0 (33.5, 44.5)	0.10
Liver diameter (cm)	20.00 (17.0, 22.3)	20.8 (18.2, 24.5)	0.05
GAHS	9.0 (8.0, 9.0)	8.0 (8.0, 9.0)	0.11
Maddrey Discriminant Function	65.1 (51.9, 81.8)	62.5 (50.4, 76.5)	0.49
MELD Score	25.0 (23.0, 27.3)	25.0 (23.0, 27.0)	0.41
*Laboratory values*			
Bilirubin (mg/dl)	22.0 (19.1, 28.9)	24.9 (21.0, 28.0)	0.36
Creatinine (mg/dl)	0.7 (0.6, 0.9)	0.6 (0.5, 0.9)	0.24
Urea (mg/dl)	21.4 (14.7, 34.2)	21.4 (15.0, 29.5)	0.76
De ritis quotient	2.9 (2.0, 4.0)	2.9 (1.8, 3.4)	0.60
AST (U/l)	121.0 (95.5, 159.5)	135.0 (84.5, 176.0)	0.72
ALT (U/l)	42.0 (29.0, 61.5)	45.0 (31.5, 69.5)	0.30
AP (U/l)	153.5 (112.5, 206.5)	194.0 (132.8, 240.3)	0.01
Protein (g/dl)	5.7 (5.2, 6.5)	5.9 (5.3, 6.6)	0.54
Albumin (g/dl)	2.7 (2.3, 3.2)	2.7 (2.4, 3.2)	0.90
Sodium (mEq/l)	135.0 (131.0, 138.0)	135.0 (132.0, 137.0)	0.94
Lactic acid (mmol/l)	1.6 (1.2, 2.2)	1.6 (1.3, 2.1)	0.85
Ammonia (µg/dl)	81.3 (63.0, 117.7)	91.1 (62.0, 112.8)	0.72
Hemoglobin (g/dl)	9.9 (8.6, 11.2)	10.6 (9.1, 11.7)	0.07
White blood cells (10^9^ /l)	11.8 (8.1, 16.7)	12.9 (8.3, 17.4)	0.45
Platelets (10^9^ /l)	159.0 (94.8, 227.0)	154.0 (105.0, 194.0)	0.94
INR	1.8 (1.5, 2.2)	1.8 (1.5, 2.0)	0.45
*Comorbidities*			
Liver cirrhosis	60 (68.2)	46 (73.0)	0.65
Diabetes mellitus	10 (11.4)	7 (11.1)	0.96
Acute kidney injury at BL	5 (5.7)	6 (9.5)	0.56
Infection at BL	25 (28.4)	19 (30.2)	0.96
*Substance use and concomitant therapeutics*			
Time since last alcohol consumption before admission (weeks)	1.0 (1.0, 3.0)	1.00 (1.0, 2.0)	0.27
Alcohol intake during follow-up	12 (13.6)	9 (14.3)	0.91
ELAD group	49 (55.7)	27 (42.9)	0.17
Pentoxifylline (within 7 days before BL)	15 (17.0)	12 (19.0)	0.92

### Incidence and severity of AKI

During the 90-day follow-up, a total of 63 patients (41.7%) developed AKI. For 40 of these patients, detailed grading of AKI severity was available. Among them, 28 patients (70%) presented with stage 1 AKI, 11 patients (27.5%) with stage 2 AKI, and 1 patient (2.5%) with stage 3 AKI at initial diagnosis. In the remaining 23 patients, AKI was reported as an adverse event without formal grading or available laboratory data. Median time to AKI was 3 days. Patients with AKI died after a median of 29 days, while death occurred after 46 days in patients without AKI (p = 0.07).

### Impact of AKI on mortality

**Fig. 2:**
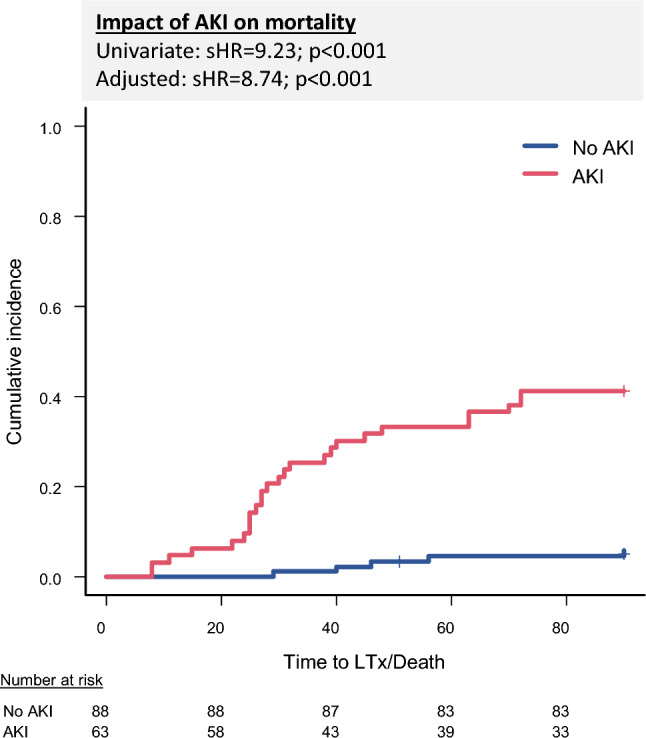
Competing risk analyses highlight a significant association between the presence of AKI and an increased mortality within the 90 days observational period. AKI: Acute kidney injury, sHR: Subdistribution hazard ratio.

Competing risk analysis demonstrated a significant association between AKI and increased mortality within 90 days (Fig. 2). In the univariate analysis, AKI was associated with a sHR of 9.23 (95% CI: 3.63–23.50; p < 0.001). This association remained robust in the multivariate model after adjustment for GAHS (sHR = 8.74; 95% CI: 3.43–22.26; p < 0.001) (Supplementary Table 4). Cox regression analysis with time-dependent covariates confirmed the significant effect of AKI on mortality, showing an HR of 12.69 in the univariate analysis (95% CI: 4.92–32.72; p < 0.001) and an HR of 12.05 in the multivariate model (95% CI: 4.62–31.08; p < 0.001) (Supplementary Table 4).

### Predictors of AKI

In competing risk regression (death and LTx as competing events), higher bilirubin levels (sHR = 1.06; 95% CI: 1.02–1.10; p = 0.004), Maddrey score (sHR = 1.02; 95% CI: 1–1.03; p = 0.014), and MELD score (sHR = 1.17; 95% CI: 1.04–1.33; p = 0.009) were significantly associated with an increased AKI risk. Conversely, corticosteroid therapy was associated with a significantly lower risk of developing AKI (sHR = 0.49; 95% CI: 0.28–0.88; p = 0.02). In multivariate competing risk analysis, only bilirubin (sHR = 1.06; 95% CI: 1.02–1.11; p = 0.003) and corticosteroid therapy (sHR = 0.47; 95% CI: 0.27–0.84; p = 0.01) remained independently associated with AKI risk. Other laboratory and clinical variables, including WBC, INR, urea, and the presence of liver cirrhosis, were not significantly associated with AKI development (Supplementary Table 5).

### Impact of corticosteroid therapy on AKI incidence

**Fig. 3:**
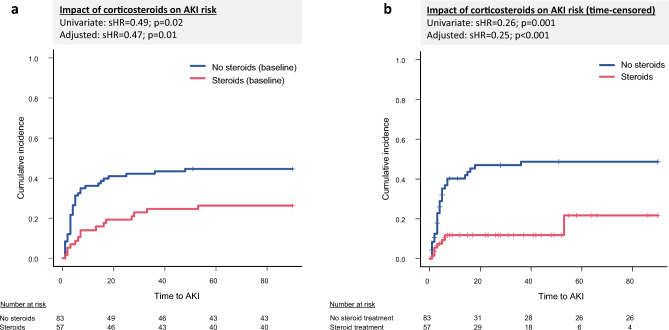
illustrates the impact of corticosteroid treatment on the risk for AKI development. In **a**, patients who received corticosteroids at baseline were compared to those without. In **b**, the patients were censored upon steroid discontinuation or upon new onset of steroid therapy. AKI: Acute kidney injury, sHR: Subdistribution hazard ratio.

The administration of corticosteroids was significantly associated with a reduced risk of developing AKI during the 90-day follow-up. In the competing risk analysis, patients receiving steroids had a significantly lower risk of AKI compared to those without steroid therapy (univariate sHR = 0.49; 95% CI: 0.28–0.88; p = 0.017). This protective effect remained robust after adjusting for bilirubin levels in the multivariate model (sHR = 0.47; 95% CI: 0.27–0.84; p = 0.01) (Table 2, Fig. 3a) and after matching of patients with and without corticosteroid intake (sHR = 0.46; 95% CI: 0.25–0.85; p = 0.01) (Supplementary Fig. 2a).

**Table 2:** Impact of corticosteroid treatment on AKI risk. Competing risk analyses unveiled a linkage between steroid intake and an ameliorated risk for AKI development. This association remained statistically significant after adjusting for Bilirubin. First, patients with steroid intake at baseline were compared to those without. In a second approach, patients were censored at time of steroid discontinuation or with the new onset of steroid therapy. CI: Confidence interval, sHR: Subdistribution hazard ratio.

	Univariate				Multivariate			
	sHR	Lower 95% CI	Upper 95% CI	p value	sHR	Lower 95% CI	Upper 95% CI	p value
Steroids	0.49	0.28	0.88	0.02	0.47	0.27	0.84	0.01
Bilirubin	1.06	1.02	1.104	0.004	1.06	1.02	1.11	0.003
Time-censored corticosteroid use								
Steroids	0.26	0.11	0.58	0.001	0.25	0.11	0.55	0.001
Bilirubin	1.06	1.02	1.11	0.006	1.07	1.02	1.11	0.004

To account for changes in steroid exposure during follow-up, a time-censored competing risk model was performed, censoring patients at the time of steroid initiation or discontinuation. Here, corticosteroid therapy remained strongly associated with a reduced AKI risk (univariate sHR = 0.26; 95% CI: 0.11–0.58; p = 0.001; multivariate sHR = 0.25; 95% CI: 0.11–0.55; p = 0.001) (Table 2, Fig. 3b). Matched analyses supported the linkage between steroid treatment and an ameliorated risk for AKI development (sHR = 0.25; 95% CI: 0.10–0.57; p = 0.001 (Supplementary Fig. 2b).

### Factors associated with AKI reversal

**Fig. 4:**
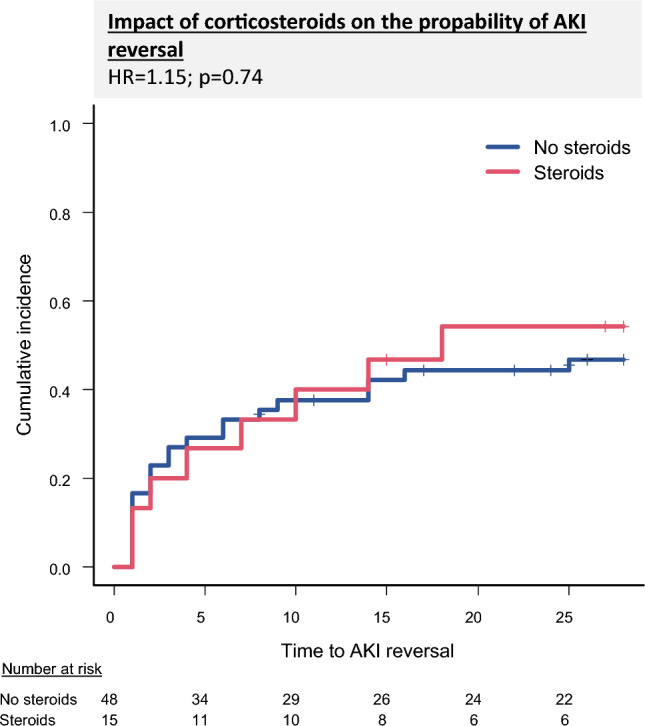
displays the influence of corticosteroid intake on AKI reversal within 28 days after AKI onset. AKI: Acute kidney injury, HR: Hazard ratio.

In Cox regression analysis, corticosteroid therapy was not significantly associated with AKI reversal (HR = 1.15; 95% CI: 0.51–2.57; p = 0.74) (Fig. 4). Higher bilirubin levels were associated with a significantly lower probability of AKI reversal (HR = 1; 95% CI: 0.99–1; p = 0.046). Furthermore, patients who received albumin therapy had a reduced likelihood of renal recovery (HR = 0.36; 95% CI: 0.18–0.74; p = 0.01). Other clinical and laboratory parameters, including WBC, INR, and the presence of liver cirrhosis, were not significantly associated with AKI reversal (Supplementary table 6).

## Discussion

AKI is a major and potentially life-threatening complication in patients with sAH. In this post-hoc analysis of 151 patients from the VTL-308 trial, we evaluated 63 cases of AKI to better characterize its incidence, clinical relevance and associated risk factors. Our findings highlight that AKI is not only frequent but also strongly associated with adverse outcomes. Despite a predominance of mild AKI stages at initial diagnosis, the development of AKI was associated with an 8- to 12-fold increased risk of death within 90 days compared to patients without AKI. This association was robust across different analytical models.

The incidence of AKI in our cohort was high, affecting 41.7% of patients within 90 days. This is consistent with previously reported prevalences of AKI in sAH in the literature ranging from 28 to 65%^5–7,14^. Whilst AKI in DC and ACLF has been extensively studied, data regarding its clinical significance and associated risk and protective factors in the context of sAH remain limited. Given this gap in literature, it is useful to interpret our sAH-findings against the broader background of existing evidence from DC and ACLF, where renal dysfunction is a well-established and major determinant of prognosis.

Regarding the incidence of AKI in ACLF, the EASL-CLIF consortium uses kidney dysfunction as a defining feature of ACLF^15^, thereby renal impairment is present in the far majority of cases. The APASL definition of ACLF does not incorporate kidney dysfunction in its definition^16^ and studies based on APASL criteria have reported renal dysfunction in 22.8%—51% of patients^17^.

Systemic inflammation is considered to be one of the key drivers in ACLF-associated AKI^18–20^. Histopathological studies have shown that the proximal tubules are the prime victim of injury in inflammation-related AKI^21^. Moreover, cholemic nephropathy is increasingly recognized as a contributing factor to renal dysfunction, particularly in patients with severe jaundice^22–24^. Similar mechanisms are involved in sAH as well and there is certainly a considerable overlap of the syndromes in routine clinical practice. However, recent data by Ma et al. suggest that the nature and implications of AKI may differ substantially in patients with and without alcohol-related liver disease^25^. Compared to patients without AH, those with underlying AH seemed to be generally younger, less often had preexisting chronic kidney disease, but presented with more severe illness, as indicated by higher MELD and CLIF-SOFA on admission. Notably, AKI phenotypes that were documented in the study by Ma et al. varied significantly: Acute tubular necrosis (ATN) was more common in patients with AH, while prerenal AKI was less frequently diagnosed. HRS-AKI, long considered the most common form of renal dysfunction in ACLF, was rarely diagnosed. These findings point toward distinct pathophysiological mechanisms in AH-related AKI. In this study, all patients had underlying cirrhosis.

When grouping sAH under the broader concept of ACLF, there is a risk of overlooking disease specific mechanisms of sAH. In contrast to DC or ACLF, sAH can occur in the absence of advanced cirrhosis and is characterized by intense systemic inflammation. Our cohort provides a unique opportunity to investigate inflammation-driven kidney injury in sAH. Key inclusion criteria required the absence of extrahepatic organ failure, including both acute and chronic kidney injury, and the absence of advanced cirrhosis. The cohort was notably young (median age 40 years), and one third of the patients showed no radiological signs of cirrhosis at baseline. In the remaining cases, advanced cirrhosis was ruled out. Imaging confirmed the absence of shrunken or nodular livers, with a mean liver diameter of 20 cm, consistent with the characteristic hepatomegaly, fibrosis and steatosis seen in sAH. All patients showed evidence of systemic inflammation, as indicated by elevated leukocyte counts.

Among the risk factors analyzed in our cohort, elevated bilirubin levels emerged as a strong predictor of poor prognosis, whereas steroid therapy was associated with a protective effect against the development of AKI.

The link between hyperbilirubinemia and kidney injury has been well-established in previous studies^24,26^. It is likely that bilirubin serves primarily as a surrogate marker of hepatocyte damage, hepatic inflammation and impaired hepatic detoxification, while other hepatotoxins, such as bile acids, may directly contribute to the development of kidney injury^27^.

In contrast, the observation that steroid therapy may exert a protective effect against AKI in sAH has not been previously reported in the literature. To our knowledge, this association was first mentioned as a secondary finding in a recent post-hoc analysis focusing on laboratory markers of AKI^14^. Our study confirms this novel association in an independent cohort and provides additional analysis supporting a potential renoprotective role of steroids in this setting.

Steroid therapy was associated with a lower risk of AKI onset but did not promote recovery once AKI had developed. This finding aligns with emerging concepts of inflammation-driven AKI, in which early injury is dominated by inflammatory and stress responses in tubular epithelial cells while structural integrity remains preserved^28,29^. During this phase, anti-inflammatory interventions may mitigate progression to irreversible damage. Once structural injury such as acute tubular necrosis has occurred, corticosteroids are unlikely to restore renal function, as they primarily modulate inflammation but do not promote cellular regeneration.

Consistent with this pathophysiological framework, higher bilirubin levels were independently associated with a reduced probability of AKI reversal. Moreover, patients receiving albumin therapy exhibited a lower likelihood of renal recovery, likely reflecting treatment selection bias, as albumin was preferentially administered to more severely ill patients. Other clinical and laboratory variables, including WBC count, INR, and presence of liver cirrhosis, were not significantly associated with AKI reversal.

The reliance on data from a single multicenter trial must be acknowledged as limitation of our study. Although post-hoc analyses might only permit restricted inferences regarding causal relationships, the use of prospectively collected data, combined with competing risk models, enhances the validity of the conclusions drawn. The overall uniform use and dosage of corticosteroids across patients who received steroid treatment enhances the adherence to current treatment guidelines and comparability between the studied patients. However, it concomitantly restricts the possibility to investigate potential impact of different steroid dosages. Regarding the number of included patients, it cannot be excluded that the sample size might have been too small to document some minor effects. However, given the strong associations between AKI and mortality risk, the limited sample size seems to be of minor relevance for the interpretation of our main and clinically important study objectives. Furthermore, information concerning AKI severity was only available for 40 of the 63 patients with AKI, reflecting an additional limitation. Histological confirmation of sAH was not required for study inclusion. However, this is analogous to the inclusion criteria of other large randomized studies, such as the STOPAH trial, as liver biopsy is not a common procedure in this patient collective^4^.

Managing complications in patients with liver diseases is of paramount importance. Our study identified a consistent protective association between corticosteroid therapy and AKI, a finding with potential therapeutic implication in this vulnerable population. Therefore, our study might contribute to the initiation of future prospective trials to further investigate the association between corticosteroids and AKI development.

## Supplementary Information

Supplementary Information 1.

Supplementary Information 2.

## Data Availability

The data are available from the corresponding author upon reasonable request.

## Abbreviations

ACLF: Acute-on-chronic liver failure
AKI: Acute kidney injury
ALT: Alanine Aminotransferase
APASL: Asian Pacific Association for the Study of the Liver
AST: Aspartate Aminotransferase
ATN: Acute tubular necrosis
CI: Confidence interval
EASL-CLIF: European Association for the Study of the Liver-Chronic Liver Failure Consortium
ELAD: Extracorporeal liver assist device
GAHS: Glasgow alcoholic hepatitis score
HR: Hazard ratio
HRS-AKI: Hepatorenal syndrome – acute kidney injury
ICA: International Club of Ascites
INR: International normalized ratio
IQR: Interquartile range
LTx: Liver transplantation
MDF: Maddrey discriminant function
MELD: Model for End-Stage Liver Disease
sAH: Severe alcohol-associated hepatitis
sCr: Serum creatinine
sHR: Subdistribution hazard ratio
SOC: Standard of care
WBC: White blood cells
