# Supplementary material for: Steroid therapy is linked to lower incidence of acute kidney injury in patients with severe alcohol-associated hepatitis
Source: Sci Rep. 2025 Dec 8;15:43271. doi: 10.1038/s41598-025-29912-4 (PMC12686481; doi:10.1038/s41598-025-29912-4)
Supplement: Supplementary file 1 — Supplementary Information 1. [file 41598_2025_29912_MOESM1_ESM.pdf]

Steroid therapy is linked to lower incidence of acute kidney injury in patients with severe alcohol-associated hepatitis

Supplementary figures

Laura Buttler[1], Jan Stange[2], Nikolaos Pyrsopoulos[3], Tarek Hassanein[4], Heiner Wedemeyer[1,5], Benjamin Maasoumy[1,5], Markus Busch[1]
on behalf of the VTL-308 study group

[1]Department of Gastroenterology, Hepatology, Infectious Diseases and Endocrinology, Hannover Medical School, Hannover, Germany
[2]Center for Extracorporeal Organ Support (CEOS), Biomedical Research Center, Department of Nephrology, University of Rostock, Rostock, Germany
[3]Liver Disease in New Jersey, NYU Grossman School of Medicine, NYU Langone Transplant Institute, New York, USA
[4]Southern California Research Center, Coronado, California, USA
[5]German Center for Infection Research (DZIF), Hannover-Braunschweig, Germany

Supplementary figure 1

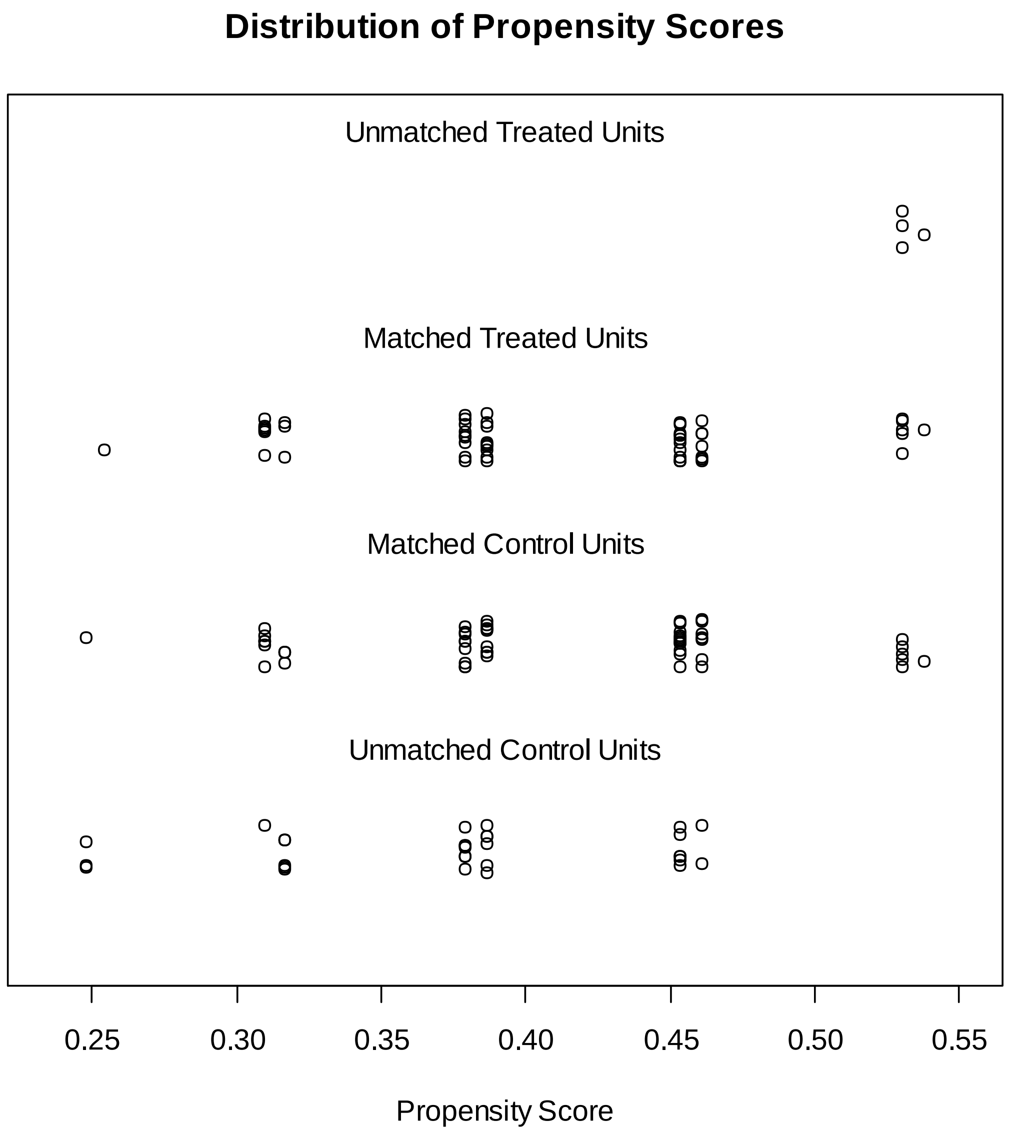

Supplementary figure 1: Jitter plot showing the distribution of propensity scores.

Supplementary figure 2

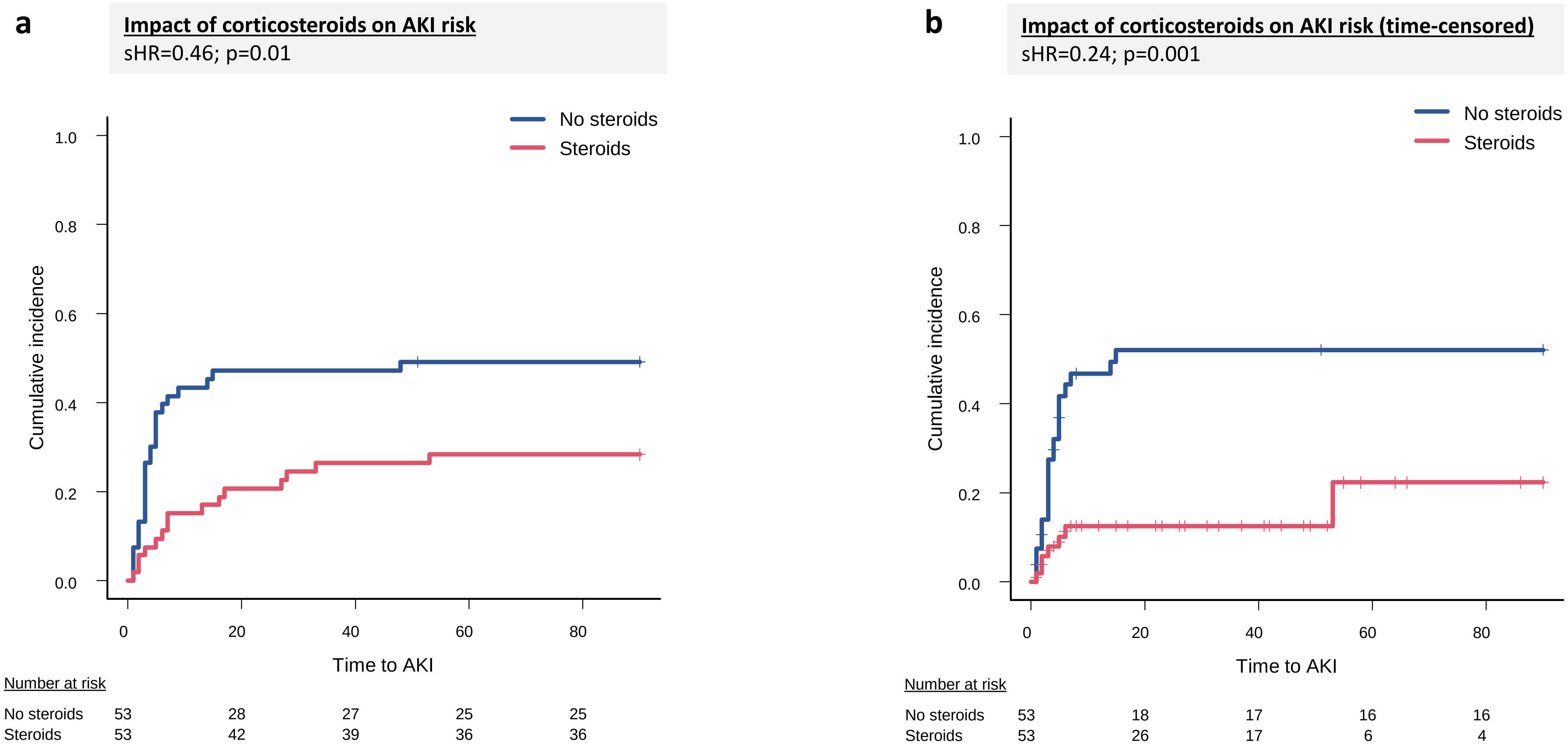

Supplementary figure 2: Supplementary figure 2 shows the impact of corticosteroid treatment on the risk for AKI development after matching patients with corticosteroid treatment with those without. AKI: Acute kidney injury, sHR: Subdistribution hazard ratio.
