# Supplementary material for: Steroid therapy is linked to lower incidence of acute kidney injury in patients with severe alcohol-associated hepatitis
Source: Sci Rep. 2025 Dec 8;15:43271. doi: 10.1038/s41598-025-29912-4 (PMC12686481; doi:10.1038/s41598-025-29912-4)
Supplement: Supplementary file 2 — Supplementary Information 2. [file 41598_2025_29912_MOESM2_ESM.pdf]

Steroid therapy is linked to lower incidence of acute kidney injury in patients with severe alcohol-associated hepatitis

- Supplementary tables –

Laura Buttler[1], Jan Stange[2], Nikolaos Pyrsopoulos[3], Tarek Hassanein[4], Heiner Wedemeyer[1,5], Benjamin Maasoumy[1,5], Markus Busch[1]

on behalf of the VTL-308 study group

[1]Department of Gastroenterology, Hepatology, Infectious Diseases and Endocrinology, Hannover Medical School, Hannover, Germany

[2]Center for Extracorporeal Organ Support (CEOS), Biomedical Research Center, Department of Nephrology, University of Rostock, Rostock, Germany

[3]Liver Disease in New Jersey, NYU Grossman School of Medicine, NYU Langone Transplant Institute, New York, USA

[4]Southern California Research Center, Coronado, California, USA

[5]German Center for Infection Research (DZIF), Hannover-Braunschweig, Germany

Supplementary table 1: Inclusion and exclusion criteria of the VTL-308 trial.

Inclusion	Exclusion
A clinical diagnosis of alcohol-induced liver decompensation (AILD)	Serum Creatinine >=1.3 mg/dL (>=115.04 umol/L)
Subjects must have severe acute alcoholic hepatitis (sAAH) diagnosed	International Normalized Ratio >2.5
Maddrey score >=32	Platelet count <40,000/mm^3
Total bilirubin >=16 mg/dL (>=273.6 umol/L)	Model for End-Stage Liver Disease score >=30
Age >=18 years	Age >=50
Written Informed Consent must be provided by the patient or a legally-authorized representative	Aspartate aminotransferase > 500 IU/L
Subject must be eligible for Standard of Care treatment as defined in the protocol	Reduction in total bilirubin of 20% or more in the previous 72 hours
	Presence of infection unresponsive to antibiotics
	Subject ventilated or intubated
	Active bleeding, major hemorrhage occurring within 48 hours prior to Randomization, or with banding of gastroesophageal varices during the 7 days immediately preceding screening
	Presence of hemodynamic instability
	Occlusive portal vein thrombosis impairing hepatopetal flow
	Bile duct obstruction
	Evidence by physical exam, history, or laboratory evaluation, of significant concomitant disease with a life expectancy of less than 3 months
	Subject has chronic end-stage renal disease requiring chronic hemodialysis for more than 8 weeks (not classified as hepatorenal syndrome)
	Subject on hemodialysis
	Clinical evidence of liver size reduction due to cirrhosis, unless Investigator interpretation otherwise and Sponsor agrees
	Subject has liver disease related to homozygous hemachromotosis, Wilson's disease, has nonalcoholic fatty liver disease, or Budd-Chiari Syndrome
	Serological evidence of active viral hepatitis A, B or C infection. If subject at risk for viral hepatitis A, B or C, and no serology is available, serologies must be obtained prior to Randomization
	Previous liver transplant
	Previous enrollment in the treatment phase of another ELAD trial
	Participation in another investigational drug, biologic, or device study within one month of enrollment, except for observational studies

	Have a Do Not Resuscitate or a Do Not Intubate (DNR/DNI) directive or any other Advanced Directive limiting Standard of Care in place
	Refusal to participate in the VTL-308E follow-up study
	Missing address for home visits
	Pregnancy as determined by serum beta-human chorionic gonadotropin results, or subjects not willing to use effective means of contraception, without history of medical or surgical sterilization

Supplementary table 2: Standardized mean differences for matching parameters before and after propensity score matching. Matching was considered to be successful with standardized mean differences between -0.1 and 0.1. GAHS: Glasgow alcoholic hepatitis score.

	**Standardized mean differences before matching**	**Standardized mean differences after matching**
**Sex**	-0.02	0.04
**GAHS**	-0.29	<0.001

Supplementary table 3: Baseline characteristics after propensity score matching. ALT: Alanine aminotransferase, AST: Aspartate aminotransferase, BL: Baseline, GAHS: Glasgow alcoholic hepatitis score, INR: International normalized ratio, MELD: Model for End-Stage Liver Disease, Y: Years.

	**No steroids**	**Steroids**	**p value***
**Number**	53 (50.0)	53 (50.0)	
**Male sex**	33 (62.3)	32 (60.4)	1.0
**Age (y)**	41.0 (34.0-45.5)	39.0 (33.0-45.0)	0.36
**Liver diameter (cm)**	20.0 (17.0-22.0)	21.4 (18.9-25.0)	0.01
**GAHS**	9.0 (8.0-9.0)	9.0 (8.0-9.0)	1.0
**Maddrey Discriminant Function**	61.8 (45.1-75.8)	63.6 (49.6-79.0)	0.78
**MELD Score**	25 (23-27)	25 (23-27)	0.79
**<u>Laboratory values</u>**			

**Bilirubin (mg/dl)**	22.0 (18.9-28.1)	24.9 (21.0-28.0)	0.33
**Creatinine (mg/dl)**	0.7 (0.6-0.9)	0.6 (0.5-0.8)	0.10
**Urea (mg/dl)**	17.6 (10.7-25.8)	21.4 (13.2-29.4)	0.27
**AST (U/l)**	139.0 (97.0-176.5)	135.0 (83.5-176.0)	0.76
**ALT (U/l)**	43.0 (28.5-67.0)	43.0 (31.0-72.5)	0.67
**INR**	1.7 (1.5-2.1)	1.8 (1.5-2.1)	0.77
**Comorbidities**			
**Liver cirrhosis**	34 (64.2)	39 (73.6)	0.36
**Diabetes mellitus**	3 (5.7)	6 (11.3)	0.51

Values stated as number (percentage) or median (interquartile range).

McNemar was used for comparison of categorial variables, Wilcoxon for continuous parameters.

Supplementary table 4: Relevance of AKI for the clinical outcome. In a first approach, competing risk analyses were conducted. In a second approach, cox proportional hazard regression treating AKI as time-dependent covariate was utilized to avoid the immortal time bias. The multivariate models incorporated the GAHS as covariable. CI: Confidence interval, GAHS: Glasgow alcoholic hepatitis score, HR: Hazard ratio, sHR: Subdistribution hazard ratio.

	**Univariate**				**Multivariate**			
	**sHR**	**Lower 95% CI**	**Upper 95% CI**	**p value**	**sHR**	**Lower 95% CI**	**Upper 95% CI**	**p value**
**Competing risk analysis - 90 days of follow-up**								
**AKI**	9.23	3.63	23.5	<0.001	8.74	3.43	22.26	<0.001
GAHS	1.40	0.99	1.98	0.06	1.27	0.85	1.91	0.25
	**HR**	**Lower 95% CI**	**Upper 95% CI**	**p value**	**HR**	**Lower 95% CI**	**Upper 95% CI**	**p value**
**Time dependent covariate - 90 days of follow-up**								
**AKI**	12.69	4.92	32.72	<0.001	12.05	4.62	31.08	<0.001
GAHS					1.47	1.00	2.16	0.05

Supplementary table 5: This table shows the results of competing risk analyses evaluating predictors for AKI development within 90 days of follow-up. ALT: Alanine aminotransferase, AST: Aspartate aminotransferase, BL: Baseline, CI: Confidence interval, ELAD: Extracorporal Liver Assist Device, GAHS: Glasgow alcoholic hepatitis score, INR: International normalized ratio, MELD: Model for End-Stage Liver Disease, SIRS: Systemic inflammatory response syndrome, sHR: Subdistribution hazard ratio.

	Univariate				Multivariate			
	sHR	Lower 95% CI	Upper 95% CI	p value	sHR	Lower 95% CI	Upper 95% CI	p value
**Age**	1.03	0.98	1.07	0.2				
**Leukocytes**	1.01	0.98	1.04	0.44				
**Urea**	1.01	1.00	1.02	0.09				
**INR**	1.8	0.89	3.66	0.1				
**Creatinine**	0.96	0.93	1.00	0.08				
**Bilirubin**	1.06	1.02	1.10	0.004	1.06	1.02	1.11	0.003
**Maddrey**	1.02	1.00	1.03	0.01				
**GAHS**	1.22	0.95	1.58	0.12				
**MELD**	1.17	1.04	1.33	0.01				
**De ritis ratio**	1.01	0.86	1.19	0.92				
**Steroids (BL)**	0.49	0.28	0.88	0.02	0.4743	0.2688	0.8371	0.01
**Pentoxifylline (BL)**	0.84	0.32	2.18	0.72				
**ELAD**	1.07	0.63	1.82	0.81				
**Antibiotics**	1.03	0.60	1.75	0.93				
**Cephalosporins**	1.85	0.90	3.80	0.10				
**Piperacillin/Tazobactam**	1.61	0.64	4.09	0.31				
**Vancomycin**	1.13	0.32	3.96	0.85				
**Diabetes**	1.52	0.71	3.26	0.28				
**Infection at baseline**	1.37	0.78	2.40	0.27				
**AST**	1.00	0.99	1.00	0.74				
**ALT**	1.01	1.00	1.01	0.16				
**Albumin**	0.81	0.53	1.51	0.68				
**Lactic acid**	1.28	0.93	1.78	0.13				

**SIRS (BL)**	0.91	0.53	1.58	0.75	
**Ascites (BL)**	1.44	0.57	3.64	0.44	
**Moderate/severe ascites (BL)**	0.89	0.51	1.56	0.68	
**Liver cirrhosis**	1.21	0.65	2.27	0.54	

Supplementary table 6: Predictors of AKI reversal. The predictive potential of relevant clinical parameters for the likelihood of AKI reversal was evaluated. ALT: Alanine aminotransferase, AST: Aspartate aminotransferase, CI: Confidence interval, GAHS: Glasgow alcoholic hepatitis score, HR: Hazard ratio.

	**Univariate**			
	**HR**	**Lower 95% CI**	**Upper 95% CI**	**p value**
**Age**	0.99	0.93	1.04	0.62
**Leukocytes**	1.01	0.98	1.04	0.53
**Urea**	0.99	0.98	1.01	0.22
**INR**	0.77	0.37	1.61	0.49
**Creatinine**	0.88	0.44	1.76	0.72
**Bilirubin**	0.997	0.994	1.00	0.046
**GAHS**	1.04	0.65	1.64	0.88
**MELD**	0.91	0.82	1.02	0.09
**De ritis ratio**	1.03	0.99	1.06	0.13
**Steroids**	1.15	0.51	2.57	0.74
**Pentoxifylline**	1.67	0.64	4.38	0.30
**ELAD**	1.21	0.49	2.96	0.68
**Albumin therapy**	0.36	0.18	0.74	0.01
**Terlipressin**	1.23	0.43	3.54	0.70
**AST**	1.00	1.00	1.001	0.39
**ALT**	1.00	0.99	1.02	0.53

**Albumin**	1.12	0.66	1.91	0.67
**Lactic acid**	1.27	0.87	1.85	0.21
**Ascites**	0.78	0.37	1.61	0.50
**Liver cirrhosis**	1.82	0.74	4.45	0.19
**Diabetes**	0.83	0.29	2.38	0.73
